# Discovering a Four-Gene Prognostic Model Based on Single-Cell Data and Gene Expression Data of Pancreatic Adenocarcinoma

**DOI:** 10.3389/fendo.2022.883548

**Published:** 2022-06-21

**Authors:** Weizhen Huang, Jun Li, Siwei Zhou, Yi Li, Xia Yuan

**Affiliations:** ^1^ The Second Department of Medical Oncology, Huizhou First Hospital, Huizhou, China; ^2^ Cancer Center, Huizhou First Hospital, Huizhou, China

**Keywords:** pancreatic adenocarcinoma, tumor microenvironment, memory B cells, immune subtype, prognostic model, bioinformatics analysis

## Abstract

**Background:**

Pancreatic cancer has a 5-year overall survival lower than 8%. Pancreatic adenocarcinoma (PAAD) is the most common type. This study attempted to explore novel molecular subtypes and a prognostic model through analyzing tumor microenvironment (TME).

**Materials and Methods:**

Single-cell RNA sequencing (scRNA-seq) data and expression profiles from public databases were downloaded. Three PAAD samples with single-cell data and 566 samples with gene expression data were included. Seurat was used to identify cell subsets. SVA merged and removed batch effects from multichip datasets. CIBERSORT was used to evaluate the components of different cells in transcriptome, ConsensusClusterPlus was used to identify molecular subtypes, and gene set enrichment analysis was used for functional enrichment analysis. LASSO Cox was performed to construct dimensionality reduction and prognosis model.

**Results:**

Memory B cells (MBCs) were identified to be significantly with PAAD prognosis. Two immune subtypes (IS1 and IS2) with distinct overall survival were constructed. Forty-one DEGs were identified between IS1 and IS2. Four prognostic genes (ANLN, ARNTL2, SERPINB5, and DKK1) were screened to develop a prognostic model. The model was effective in classifying samples into high-risk and low-risk groups with distinct prognosis. Three subgroups of MBCs were identified, where MBC_0 and MBC_1 were differentially distributed between IS1 and IS2, high-risk and low-risk groups.

**Conclusions:**

MBCs were closely involved in PAAD progression, especially MBC_0 and MBC_1 subgroups. The four-gene prognostic model was predictive of overall survival and could guide immunotherapy for patients with PAAD.

## Introduction

Pancreatic cancer has high death rate, and pancreatic adenocarcinoma (PAAD) is the most common pathological type. According to global cancer statistics, in 2020, 495,773 new cases of pancreatic cancer were diagnosed and 466,003 deaths occurred (1). However, the incidence varied greatly among different regions. Age-standardized rate was the highest in Europe and North America but the lowest in Africa and South Central Asia (2). Significant difference of incidence is also observed between developed countries and developing countries (3). Smoking, alcohol, obesity, and dietary factors are main risk factors contributing to the development of pancreatic cancer and its unfavorable survival (4). New diagnosed patients are common at advanced stage, resulting in a low overall survival rate of only 8% (5). Therefore, early diagnosis for pancreatic cancer is required for improving prognosis.

Tumor microenvironment (TME) plays a critical role in cancer development. The composition and distribution of different immune cells affects anti-tumor immune response and the formation of immune escape TME. Tumor-associated macrophages (TAMs), regulatory T cells (Tregs), and myeloid-derived suppressor cells (MDSCs) are major immunosuppressive cells helping tumor cells to escape immune capture. Immunosuppressive TME is involved in metastasis through activating oncogenic pathways such as angiogenesis, epithelial–mesenchymal transition (EMT), and transforming growth factor (TGF)-β signaling pathways in pancreatic cancer (6). Targeting TME is considered as a promising immunotherapy for cancer treatment. Programmed cell death protein-1 (PD-1) and cytotoxic T-lymphocyte–associated protein 4 (CTLA-4) are two important immune checkpoints that can impede anti-tumor response when combined with their receptors. The inhibitors of PD-1 and CTLA-4 could activate immune response of tumor cells in clinical trials of different cancer types, including pancreatic cancer (7). However, not all patients can benefit from immune checkpoint blockade (ICB). Further understanding of TME and molecular features in pancreatic cancer is needed to facilitate the exploration of new therapeutic drugs.

Single-cell sequencing technology facilitates a deep excavation of molecular data of TME. In this study, we introduced single-cell RNA sequencing (Single-cell RNA sequencing) data from public database and applied single-cell analysis to screen valuable information. We found that a group of immune cells, memory B cells (MBCs), was able to serve as molecular features to classify patients with PAAD into different molecular subtypes. On the basis of the markers of MBCs, we identified four prognostic genes and constructed a prognostic model that could predict overall survival for patients with PAAD. Importantly, the prognostic model was able to identify patients who may be more sensitive to ICB therapy.

## Materials and Methods

### Data Source

For the workflow of this study, see **Supplementary Figure S1A**. scRNA-seq data of normal and PAAD samples were downloaded from Gene Expression Omnibus (GEO) database (https://www.ncbi.nlm.nih.gov/geo/). Expression profiles of normal and tumor samples were downloaded from GEO, The Cancer Genome Atlas (TCGA) database (https://portal.gdc.cancer.gov/), and International Cancer Genome Consortium (ICGC) database (https://dcc.icgc.org/).

### Data Preprocessing

GSE165399 (8) cohort contained scRNA-seq data of one normal sample (GSM5032773) and two tumor samples (GSM5032771 and GSM5032772). Seurat R package was employed to preprocess single-cell data (9). Data were first screened under condition that each gene expressed at least in three cells and each cell expressed at least 250 genes. Then, “PercentageFeatureSet” function was conducted to calculate the percentage of mitochondria and rRNA. Finally, single cells were filtered under the standards that each cell expressed 500–6,000 genes, mitochondria percentage was less than 35% and unique molecular identifiers (UMI) of each cell was over than 1,000. Quality control of single-cell data before and after preprocessing was shown (**Supplementary Figures S1B, C** **,S2A**). Log-normalization was performed to normalize data of three samples. “FindVariableFeatures” function was conducted to excavate highly variable genes. Then, “FindIntegrationAnchors” was used to remove batch effects, and “IntegrateData” function was performed to combine data. Next, “ScaleData” function was used to scale data, and principle component analysis was performed to reduce data dimensionality (**Supplementary Figures S2B, C**).

For GSE21501 (10), GSE28735 (11), GSE57495 (12), GSE62452 (13), and GSE85916 cohorts, samples without survival status or survival time were excluded. To combine five cohorts, limma (14) and sva R packages were used to remove batch effects and normalize the data (named as GEO cohort) (**Supplementary Figures S2D, E**). A total of 320 PAAD samples and 16,466 genes were remained. For TCGA-PAAD and ICGC-AU cohort, samples without survival time or survival status were removed. Finally, 156 and 90 tumor samples remained in TCGA-PAAD and ICGC-AU cohort, respectively. The sample clinical characteristics of each dataset were in **Supplementary Table S1**.

### Single-Cell Annotation

First, “FindNeighbors” and “FindClusters” function in Seurat R package were performed to cluster single cells under dim = 30 and resolution = 0.5. Cells were clustered into different subgroups. Markers of different immune cells were obtained from previous research (15). Single-sample gene set enrichment analysis (ssGSEA) was conducted to calculate enrichment score of immune cells and annotate subgroups. Marker genes of different cell types related to pancreatic tissue were obtained from CellMarker (http://biocc.hrbmu.edu.cn/CellMarker/) (16). The top five enriched marker genes of each subgroup were identified using “FindAllMarkers” function under logfc = 0.5 and Minpct = 0.35 (*P* < 0.05).

### Gene Set Enrichment Analysis

Gene set enrichment analysis (GSEA) calculates enrichment score of genes, cells, or signatures based on expression profiles and is widely implemented in analyzing cancer data (17). The strength of the method is to interpret biological meaning such as functional pathways and biological process based on a series of gene sets. GSEA was performed to assess the enrichment of hallmark pathways based on a gene set “h.all.v7.4.symbols.gmt” downloaded from Molecular Signatures Database (MSigDB, https://www.gsea-msigdb.org/gsea/msigdb/). SsGSEA, which is developed based on GSEA, allows a calculation of enrichment score for each sample (18). We used ssGSEA to assess the enrichment score of 10 oncogenic pathways (19).

### Unsupervised Consensus Clustering

Unsupervised consensus clustering is a useful method to discover biological characteristics in cancer study. We applied ConsensusClusterPlus R package (20) to perform consensus clustering for samples in TCGA-PAAD cohort based on markers of MBCs. Expression data of markers were normalized. partitioning around medoids (PAM) algorithm was conducted, and “Canberra” was used as measurement distance. Five hundred bootstraps were conducted with each bootstrap containing 80% samples of TCGA-PAAD cohort. Cluster number k was set from 2 to 10. Cumulative distribution function (CDF) and area under CDF curve were used to confirm the optimal cluster number.

### Identifying Differential Expressed Genes Between Two Subtypes

Limma R package was employed to identify differential expressed genes (DEGs) between different subtypes (21). False discovery rate (FDR < 0.05) and |log2(fold change(FC))| > 1 were set to screen DEGs (both upregulated and downregulated genes).

### Constructing a Prognostic Model

TCGA-PAAD cohort served as training cohort, and GEO and ICGC-AU cohorts served as validation cohorts. Univariate Cox regression analysis in survival R package was employed to screen prognostic genes (*P* < 0.05) in TCGA-PAAD cohort. Next, least absolute shrinkage and selection operator (LASSO) Cox regression analysis in glmnet R package was performed to decrease the number of genes (22). Ten-fold cross-validation was conducted to validate the prognostic model. Receiver operating characteristic (ROC) curve analysis in timeROC R package was performed to evaluate the effectiveness of the prognostic model (23).

### Tumor Immune Dysfunction and Exclusion Analysis

To predict the response to ICB, tumor immune dysfunction and exclusion (TIDE) analysis (http://tide.dfci.harvard.edu/) was introduced (24). Signatures, including T cell dysfunction, T cell exclusion, and immunosuppressive cells, were used as a basis to calculate enrichment score for high-risk and low-risk groups. TIDE analysis was effective in predicting the mechanism of immune escape within TME for various cancer types.

### CIBERSORT Analysis

CIBERSORT (http://cibersort.stanford.edu/) was applied to evaluate the enrichment of 22 immune cells (25). The CIBERSORT tool could estimate the proportion of immune cells in TME based on gene expression data. In this study, we applied CIBERSORT to predict the enrichment score of different MBC subgroups.

### ReactomeGSA for Analyzing Single-Cell Data

To analyze the function of MBCs, Reactome database (https://reactome.org/) and ReactomeGSA were introduced (26). ReactomeGSA tool can be linked to Reactome database and enable assessment of functional pathways on multi-omics. The top 20 differentially enriched pathways were visualized.

### Statistical Analysis

All statistical analysis was performed in R software (v4.1.0). Student *t*-test was conducted between the two groups. ANOVA was conducted among three or more than three groups. Log-rank test was performed in Kaplan–Meier survival analysis. Parameters were default if there was no indication. *P* < 0.05 was considered as significant. ns, no significant, *P < 0.05, **P < 0.01, ***P < 0.001, and ****P < 0.0001.

## Results

### Defining Cells in PAAD Single-Cell Data

**Figure 1:**
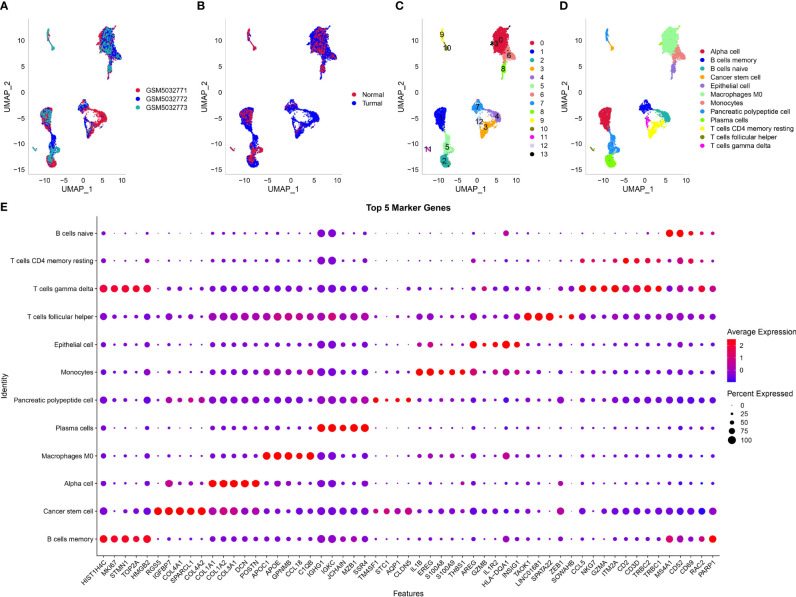
Dimensionality reduction and subgrouping of single cells. **(A)** UMAP plot of three samples including GSM5032771, GSM5032772, and GSM5032773. **(B)** UMAP plot of normal and tumoral samples. **(C)** UMAP plot of cell subgrouping. **(D)** UMAP plot of cell type definition. **(E)** The top five enriched markers of 12 cell types. Horizontal axis indicated markers and vertical axis indicated cell types.

The scRNA-seq data of three samples in GSE165399 dataset were preprocessed to screen valid data (**Supplementary Figures S1B, C** **,S2A**). After the screening, one gene expressed at least in three cells and one cell expressed at least 500 genes. The mitochondrial percent was lower than 35%, and UMI of each cell was more than 1,000. Then, the screening data were normalized, and three samples were combined to remove batch effects. Principle component analysis was applied to diminish dimensionality (**Supplementary Figures S2B, C**). Two-dimensional scRNA-seq data of single cells were clustered using UMAP, and 14 clusters were generated (**Figures 1A–C**). Compared to the normal sample (GSM5032773), tumor samples had an obviously different distribution of cells, suggesting that normal and tumor samples possibly had different cell types. According to the markers of 22 immune cells from a previous study (15), we annotated 14 subgroups, and finally, 12 cell types were identified (**Figure 1D**). The top five DEGs of 12 cell types were screened (*P* < 0.05, **Figure 1E**).

### Identifying Cell Types Associated With PAAD Prognosis

**Figure 2:**
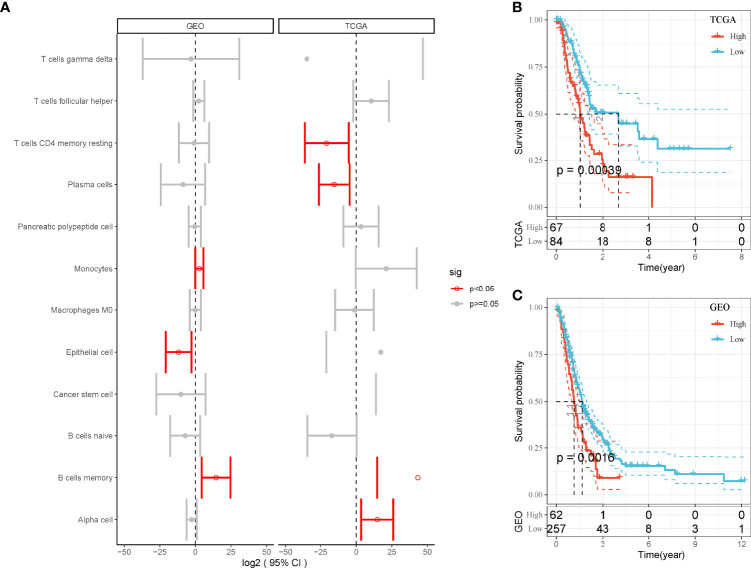
The relation between enrichment of different cell types and PAAD prognosis. **(A)** Forest plot of different cell types in the relation to prognosis in TCGA and GEO cohorts. Red indicates *P* < 0.05. **(B, C)** Kaplan–Meier survival plots of high and low enrichment of MBCs in TCGA-PAAD **(A)** and GEO **(B)** cohorts.

Then, we used the screened DEGs of 12 cell types to calculate enrichment score of each sample in TCGA and GEO cohorts. Univariate Cox regression analysis revealed that four and three cell types were associated with PAAD prognosis in TCGA and GEO, respectively (*P* < 0.05, **Figure 2A**). However, only MBCs were related to prognosis in both two cohorts. Survival analysis showed that the enrichment of MBCs was significantly associated with PAAD overall survival in the two cohorts (*P* < 0.01, **Figures 2B, C**). Low enrichment of MBCs had more favorable prognosis than high enrichment of MBCs.

### Constructing Molecular Subtypes Based on Markers of Memory B Cells

**Figure 3:**
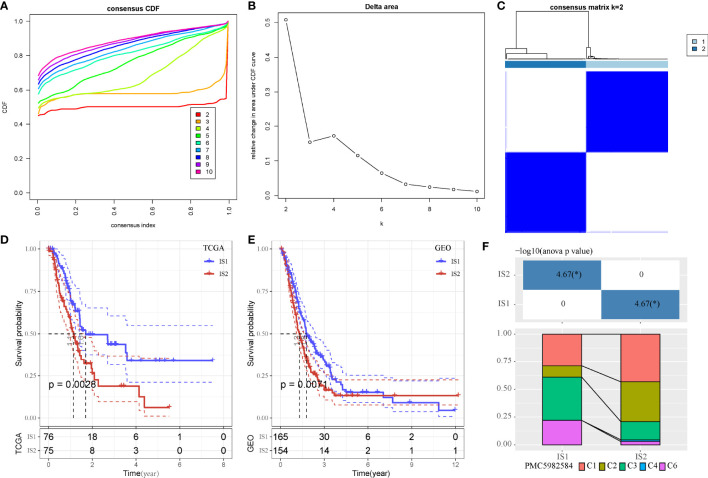
Unsupervised consensus clustering of PAAD samples based on markers of MBCs in TCGA-PAAD cohort. **(A, B)** Consensus CDF curve and area under CDF curve when k = 2–10. **(C)** Consensus matrix when k = 2. **(D, E)** Kaplan–Meier survival plot of IS1 and IS2 groups in TCGA-PAAD and GEO cohorts. Log-rank test was conducted. CDF, cumulative distribution function. **(F)** Intersection between two molecular subtypes and the previous six immune subtypes.*P < 0.05.

As we identified that MBCs were an important cell groups in PAAD, we considered that the expression of their markers were associated with prognosis. Therefore, on the basis of 107 markers of MBCs, unsupervised consensus clustering was conducted to construct molecular subtypes. According to CDF curve and delta area under CDF curve (**Figures 3A, B**), cluster number k = 2 was determined to classify PAAD into two immune subtypes (IS1 and IS2, **Figure 3C**). Survival analysis showed that IS1 had better overall survival than IS2 in both two cohorts (*P* < 0.01, **Figures 3D, E**). Six types of immune subtypes were obtained from previous studies (27), namely, C1 (wound healing), C2 (inf-r dominant), C3 (inflammation), C4 (lymphocyte depletion), C5 (immunological silencing), and C6 (TGF-β dominant). Comparison of the relationship between the two molecular subtypes and these six types of immune cell infiltration showed that IS1 mainly accounted for a large proportion than C3 and C6 and that IS2 mainly enriched with C1 and C2. There were significant distribution differences between them (p < 0.01, **Figure 3F**). In the relation between subtypes and other clinical information, subtypes were significantly associated with survival status and grade (**Supplementary Figure S3**). Dead samples were more enriched in IS2, and grade 1 was more distributed in IS1. In addition, IS2 had obviously higher proportion of high enrichment of MBCs (**Supplementary Figure S3I**), which was consistent with previous result that high enrichment of MBCs had unfavorable prognosis.

### Tumor-Related Pathways Were More Enriched in IS2

**Figure 4:**
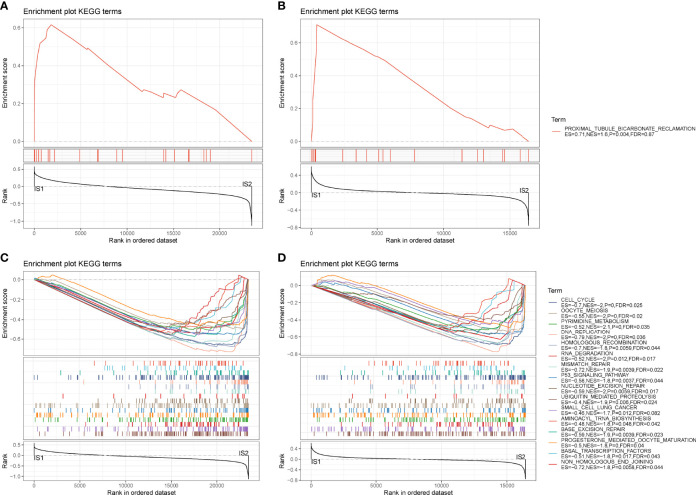
GSEA of hallmark pathways in TCGA-PAAD and GEO cohorts. **(A, B)** Enriched pathways of IS1 in TCGA-PAAD **(A)** and GEO **(B)** cohorts. **(C, D)** Enriched pathways of IS2 in TCGA-PAAD **(C)** and GEO **(D)** cohorts.

Next, we analyzed hallmark pathways of the two subtypes in two cohorts. In TCGA cohort, only one pathway was enriched in IS1, whereas 37 pathways were enriched in IS2. In GEO cohort, six pathways were enriched in IS1, and 18 pathways were enriched in IS2. Comparison of enriched pathways in two cohorts demonstrated that proximal tubule bicarbonate reclamation was enriched in IS1 in both two cohorts (**Figures 4A, B**). Eighteen pathways were enriched in IS2 in both cohorts, such as p53 signaling pathway, cell cycle, DNA replication, small cell lung cancer, and mismatch repair (**Figures 4C, D**). The results showed that tumor-related pathways were more activated in IS2, which may contribute to its worse prognosis.

### The Relation Between DEGs and Memory B Cells

**Figure 5:**
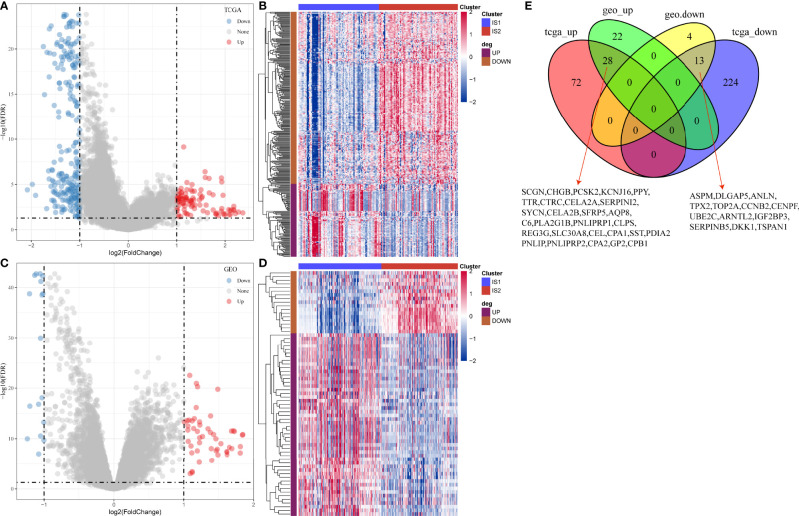
Differential analysis between IS1 and IS2. **(A)** Volcano plot of DEGs in TCGA-PAAD cohort. **(B)** Unsupervised consensus clustering for TCGA-PAAD samples based on DEGs. **(C)** Volcano plot of DEGs in GEO cohort. **(D)** Unsupervised consensus clustering for GEO samples based on DEGs. **(E)** Venn plot of upregulated and downregulated genes in TCGA-PAAD and GEO cohorts.

**Figure 6:**
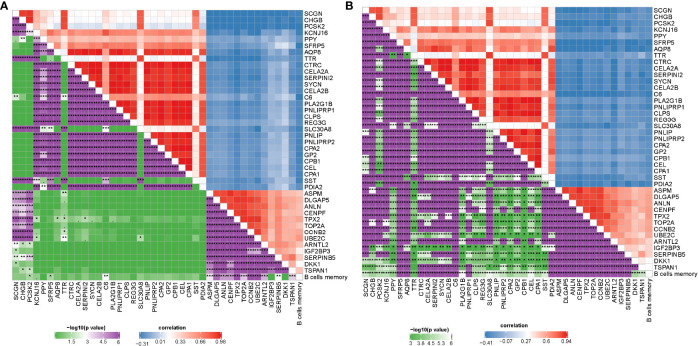
Pearson correlation analysis between MBCs and 41 key DEGs in TCGA-PAAD **(A)** and GEO **(B)** cohorts. Blue indicates negative correlation and red indicates positive correlation. *P < 0.05, **P < 0.01, and ***P < 0.001.

Gene expression profiles between IS1 and IS2 were compared to screen DEGs. In TCGA cohort, 100 upregulated and 237 downregulated genes were identified from IS1 (FDR < 0.05, |log2(FC)| > 1; **Figures 5A, B**). In GEO cohort, 50 upregulated and 17 downregulated genes were identified from IS1 (FDR < 0.05, |log2(FC)| > 1; **Figures 5C, D**). We found that 28 DEGs were upregulated and 13 DEGs were downregulated in IS1 in both two cohorts (**Figure 5E**). Furthermore, we assessed the correlation between the identified DEGs and MBCs. The results showed that 13 downregulated DEGs were positively correlated with the enrichment of MBCs, and 28 upregulated DEGs were negatively correlated with MBCs (**Figure 6**), suggesting that these DEGs were possibly involved in the PAAD development.

### Establishing a Prognostic Model Based on DEGs

As we identified 41 DEGs associated with MBCs, a prognostic model was established based on them. Univariate Cox regression analysis was applied to these DEGs in TCGA cohort, and 16 DEGs were screened to be associated with prognosis. To further decrease the number of genes, we performed LASSO Cox regression analysis. The coefficients of DEGs close to zero showed an increasing lambda value (**Supplementary Figure S4A**). Ten-fold cross-validation calculated the confidence interval of each lambda value (**Supplementary Figure S4B**). When lambda = 0.0661, the model reached the optimal. Finally, four genes were remained, including ANLN, ARNTL2, SERPINB5, and DKK1. The four-gene prognostic model was defined as risk score = 0.294*ANLN + 0.155*ARNTL2 + 0.138*SERPINB5 + 0.058*DKK1.

**Figure 7:**
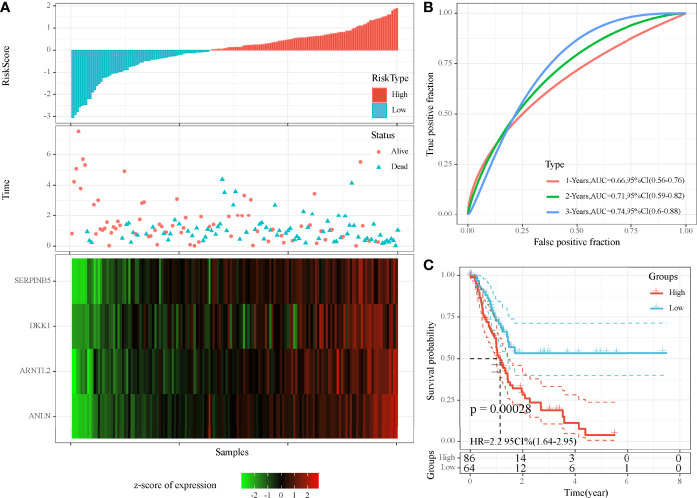
Evaluation of the four-gene prognostic model in TCGA-PAAD cohort. **(A)** The distribution of PAAD samples and expression of prognostic genes ranking by risk score. **(B)** ROC curve of the prognostic model in predicting 1-, 3-, and 5-year overall survival. **(C)** Kaplan–Meier survival plot of high-risk and low-risk groups. Log-rank test was conducted.

Risk score was calculated for each sample in TCGA cohort. Samples were divided into high-risk and low-risk groups, according to the cut-off of z-score = 0. High-risk group had more dead samples than low-risk group (**Figure 7A**). Four genes were all high-expressed in high-risk group, compared to low-risk group. ROC analysis revealed that the prognostic model had the strongest performance in predicting 5-year overall survival (AUC = 0.74, 95% CI = 0.60–0.88; **Figure 7B**). Survival analysis showed differential overall survival of two groups (*P* = 0.00028, **Figure 7C**). In another two independent cohorts (GEO and ICGC), similar results were observed, and samples could be significantly classified into high-risk and low-risk groups (**Supplementary Figures S5** **,S6**).

### Tumor Microenvironment of High-Risk and Low-Risk Groups

**Figure 8:**
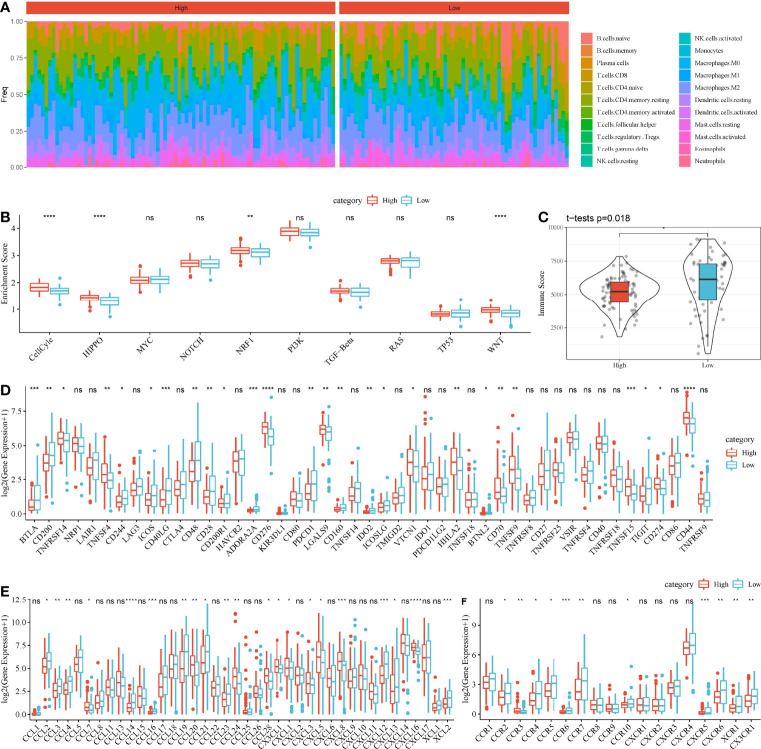
Comparison of TME between high-risk and low-risk groups in TCGA-PAAD cohort. **(A)** A heatmap describing distribution of 22 immune cells in high-risk and low-risk groups. **(B)** Enrichment score of 10 oncogenic pathways in high-risk and low-risk groups. **(C)** Immune score of high-risk and low-risk groups. **(D–F)** Expression of immune checkpoints **(D)**, chemokines **(E)**, and chemokine receptors **(F)** in two groups. Student *t*-test was performed between two groups. ns, no significance. *P < 0.05, **P < 0.01, ***P < 0.001, and ****P < 0.0001.

Then, we assessed the distribution of 22 immune cells in high-risk and low-risk groups. Macrophages and CD4 T cells contributed a large proportion in two groups (**Figure 8A**). In addition, we calculated the enrichment score of 10 oncogenic pathways, and 4 of 10 pathways were differentially enriched between the two groups (*P* < 0.01, **Figure 8B**). Cell cycle, Hippo signaling, NRF1, and WNT signaling pathways were more activated in high-risk group, indicating that these pathways were possibly involved in the PAAD development. ESTIMATE analysis revealed that low-risk group had higher immune infiltration than high-risk group (*P* = 0.018, **Figure 8C**), indicating that immune infiltration degree may affect the prognosis. To further understand the TME of two groups, we analyzed the expression of immune checkpoints, chemokines, and chemokine receptors. The data revealed that 26 of 47 immune checkpoints such as LAG3, CTLA4, PDCD1, and CD274 were differentially expressed between high-risk and low-risk groups (*P* < 0.05, **Figure 8D**). Twenty-one of 44 chemokines and 11 of 18 chemokine receptors were differentially expressed between the two groups (*P* < 0.05, **Figures 8E, F**). In GEO cohort, similar results were observed that high-risk group had higher immune infiltration than low-risk group (**Supplementary Figure S7**). Cell cycle and Hippo signaling pathway were also more activated in high-risk group. The above results indicated that high-risk and low-risk groups had different TME that may lead to different immune response.

### Differential Immune Response to Immunotherapy of Two Groups

**Figure 9:**
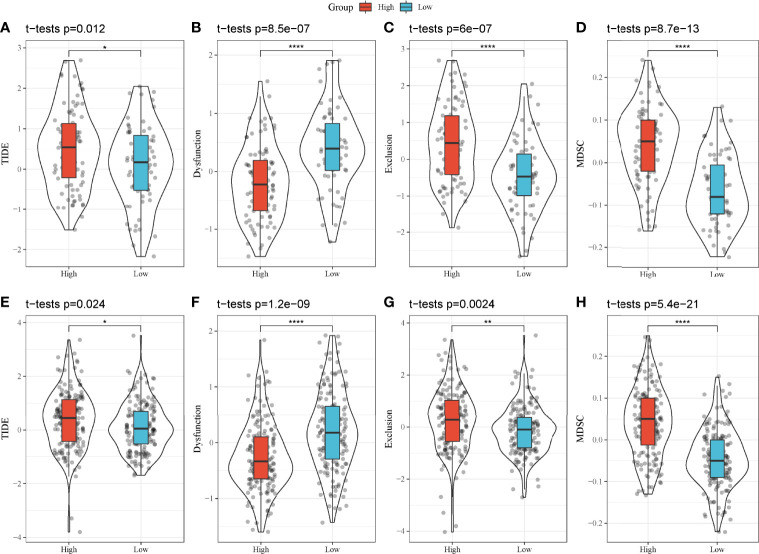
TIDE analysis of high-risk and low-risk groups. **(A–H)** Comparison of TIDE score **(A, E)**, T cell dysfunction **(B, F)**, T cell exclusion **(C, G)** and enrichment score of MDSCs **(D, H)** between high-risk and low-risk groups in TCGA-PAAD **(A–D)** and GEO **(E–H)** cohorts. *P < 0.05, **P < 0.01, and ****P < 0.0001.

As high-risk and low-risk groups displayed distinct TME and expression of immune checkpoints, we speculated that they may have different response to ICB. Therefore, TIDE analysis was applied to calculate TIDE score for two groups in TCGA and GEO cohorts. In TCGA cohort, high-risk group had higher TIDE score than low-risk group, suggesting that low-risk group was more sensitive to ICB (P = 0.012, **Figure 9A**). In addition, high-risk group had lower score of T cell dysfunction but higher score of T cell exclusion (P < 0.0001, **Figures 9B, C**), indicating the different mechanism of immune escape of two groups. Moreover, MDSCs, an immunosuppressive cell type, were highly enriched in high-risk group (P < 0.0001, **Figure 9D**). In GEO cohort, the same results were obtained (**Figures 9E–H**), demonstrating that high-risk and low-risk groups had different immune response to immunotherapy. The prognostic model associating with MBCs was robust in predicting the response to immunotherapy. In addition, we also compared the relationship between IS1-2 and existing molecular subtypes and patients in high-risk and low-risk groups. It can be observed that patients in high-risk group mainly came from IS2, C1 and C2 immune subtypes, whereas patients in low-risk group mainly came from IS1, C3 and C6 immune subtypes (**Supplementary Figure S8A**). By mapping the four key genes into the string database, it can be observed that there was no direct interaction between them, which suggested that these genes may each perform different functions (**Supplementary Figure S8B**). Analysis on the relationship between the expression of these genes and MBCs showed that ANLN had a significant positive correlation with MBCs, and ARNTL2 and SERPINB5 had a significant negative correlation with MBCs (**Supplementary Figure S8C**).

### Identifying Subgroups of MBCs Related to PAAD Prognosis

**Figure 10:**
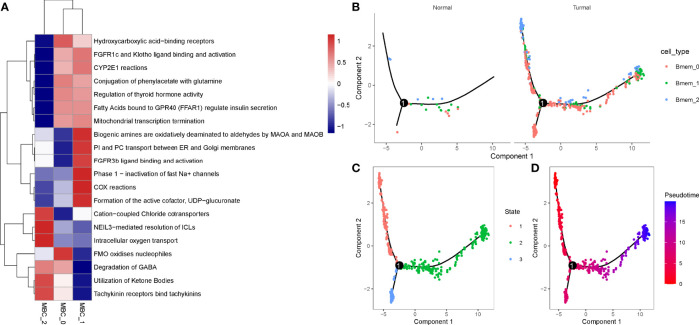
Identification of three MBC subgroups. **(A)** The top 20 enriched pathways of three MBC subgroups. Blue indicates low enrichment and red indicates high enrichment. **(B)** The distribution of MBC_0, MBC_1, and MBC_2 in normal and tumor samples. **(C, D)** Cell trajectory of state1, state2, and state3. Red to blue indicates pseudotime from early to late.

In the previous section, we found that MBCs were significantly associated with PAAD prognosis. To further evaluate the function of MBCs in PAAD development, we used unsupervised consensus clustering for 554 MBCs based on markers of MBCs. MBCs were classed into three subgroups (MBC_0, MBC_1, and MBC_2). Three subgroups expressed different markers. MBC_0 only expressed FCGR2A; MBC_1 only expressed FCGR2A, VSIR, and CXCL1; and MBC_2 only expressed CD40, CDK2, LTB, and CXCL16 (**Supplementary Figure S9A**). The three subgroups had distinct enrichment on the top 20 enriched pathways, possibly indicating different function of them (**Figure 10A**).

To understand their development and distribution, we performed monocle to reveal the cell trajectory of the three subgroups (**Figures 10B–D**). All three classes of MBCs enriched in tumor cells and slightly existed in normal cells (**Figure 10B**). State 1 located in the early pseudotime and state 3 located in the late pseudotime (**Figures 10C, D**). Of the distribution of three classes of MBCs, MBC_2 was more enriched in the early pseudotime, whereas MBC_1 majorly located in the late pseudotime (**Supplementary Figure S9B**). The expression trajectory of seven markers also obviously varied by pseudotime (**Supplementary Figure S9C**).

**Figure 11:**
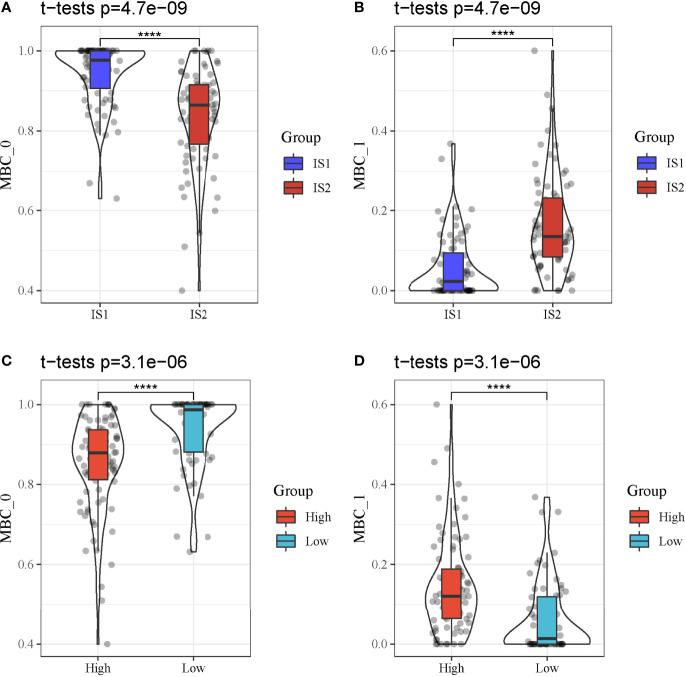
MBC enrichment of different groups in TCGA-PAAD cohort. **(A, B)** Comparison of the enrichment of MBC_0 and MBC_1 between IS1 and IS2. **(C, D)** Comparison of the enrichment of MBC_0 and MBC_1 between high-risk and low-risk groups. ****P < 0.0001.

We further analyzed whether the two types of classes (IS1 and IS2, high-risk and low-risk groups) had a difference on the distribution of different MBCs using CIBERSORT. In TCGA-PAAD dataset, only MBC_0 and MBC_1 were observed. We found a significant difference of both MBC_0 and MBC_1 enrichment between IS1 and IS2 (*P* < 0.0001, **Figures 11A, B**). Specifically, IS1 had higher enrichment of MBC_0 but lower enrichment of MBC_1, suggesting that MBC_0 may be a protective factor of PAAD prognosis. The supposal was further illustrated in high-risk and low-risk groups, as low-risk group had a higher proportion of MBC_0 compared with high-risk group (*P* < 0.0001, **Figure 11C**). However, MBC_1 was highly enriched in high-risk group (*P* < 0.0001, **Figure 11D**), which was consistent with the above results.

## Discussion

On the basis of the analysis of single-cell data, we discovered that MBCs were significantly associated with PAAD prognosis. Therefore, we constructed two molecular subtypes based on the markers of MBCs. IS1 and IS2 showed differential overall survival and clinical features, supporting that MBCs played an important role in PAAD development. To identify functional pathways that may be involved in prognosis, we analyzed the enriched pathways in IS1 and IS2 through GSEA. Tumor-related pathways such as cell cycle, DNA replication, mismatch repair, and p53 signaling pathway were highly enriched in IS2 group, suggesting that these pathways may result in worse prognosis of IS2.

A total of 41 DEGs were found between IS1 and IS2, and they were all observed to be positively or negatively associated with the enrichment of MBCs. It could be speculated that these DEGs were possibly involved in PAAD development and MBC regulation. With this hypothesis, we screened four prognostic genes (ANLN, ARNTL2, SERPINB5, and DKK1) based on 41 DEGs and constructed a four-gene prognostic model. According to the expression of four genes, risk score was calculated for each sample. PAAD samples were divided into high-risk and low-risk groups with distinct overall survival, which demonstrated that four genes were involved in cancer progression. It was worth mentioning that the prognostic relationship of these four genes in pancancer can be assessed by SangerBox online analysis platform (http://vip.sangerbox.com). We observed that these genes not only were significantly related to prognosis in pancreatic cancer but also were associated with poor prognosis in many tumors, especially lung cancer. These genes showed significant prognostic differences in lung cancer. Moreover, ANLN, SERPINB5, and ARNTL2 were also associated with poor prognosis of RCC. DDK1 and ARNTL2 were significantly associated with poor prognosis of low-grade gliomas (**Supplementary Figures S10A–D**). Previous studies have reported that these four genes biomarkers for predicting prognosis in various cancer types. Especially, DKK1 was widely reported to participate cancer development and metastasis. In breast cancer, DKK1 stimulates the metastasis of breast-to-bone through regulating WNT signaling pathway (28). However, DKK1 inhibits lung metastasis through suppressing WNT/Ca2^+^-CaMKII-NF-κB signaling, indicating a dual role of DKK1 in the metastasis of breast cancer (28). DKK1 is commonly overexpressed in many cancer types. Betella et al. proposed that DKK1 overexpression may contribute to exhaustion of effective T cells and advanced clinical stages and unfavorable prognosis in ovarian cancer (29). In our study, DKK1 was also higher-expressed in high-risk group. Moreover, a strong correlation was also found between DKK1 and MDSCs (30), where DKK1 targeted β-catenin in MDSCs in pancreatic cancer. High-risk group had a higher infiltration of MDSCs, indicating that DKK1 may have an immunomodulatory role by targeting MDSCs.

ARNTL2 was identified as a potential biomarker to predict cancer progression of colorectal cancer (31). In clear cell renal cell carcinoma, high expression of ARNTL2 is correlated with worse overall survival (32), which is consistent with our research. In addition, the group presenting high expression of ARNTL2 manifested high immune infiltration and high expression of immune checkpoints such as PD-L1 and CTLA4 (32). However, our result showed that high-risk group with high ARNTL2 expression had lower immune infiltration and lower expression of immune checkpoints, suggesting that ARNTL2 may function differentially across cancer types.

ANLN was considered to play an oncogenic role in cancer development. Zhou et al. demonstrated that knockdown of ANLN in breast cancer cell lines inhibits the proliferation of cancer cells and blocked cell cycle progression (33). Wang et al. found that ANLN expressed was significantly upregulated in pancreatic cancer, and its downregulation greatly suppresses cell proliferation and migration (34), which illustrated the prognostic value of ANLN in pancreatic cancer.

SERPINB5 has both suppressive and promotive function on cancer progression, according to previous research. In colorectal cancer, SERPINB5 overexpression is associated with poor overall survival and progression-free survival (35). Mardin et al. illustrated that upregulated SERPINB5 expression was correlated with increased metastasis resulted from SERPINB5 methylation in pancreatic ductal adenocarcinoma (36). The above observations provided evidence that SERPINB5 can serve as a prognostic biomarker for pancreatic cancer.

Overall, these four prognostic genes identified in our research have been reported to be involved in cancer development. They all could serve as prognostic biomarkers in pancreatic cancer. Our study constructed the four-gene prognostic signature that was more accurate to predict prognosis. In addition, TIDE analysis proved that the signature has the potential to guide ICB and that patients with PAAD could benefit more from personalized therapy.

Furthermore, we identified three subgroups of MBCs that had differential molecular features. MBC_0 and MBC_1 had differential enrichment in IS1 and IS2, high-risk and low-risk groups. Although we found that higher enrichment of MBCs was associated with more favorable prognosis, it was not applicable to all MBCs. MBC_0 was identified as a group of protective cells for inhibiting cancer progression as its higher enrichment in IS1 and low-risk group. However, further assessment and experiments are needed to further analyze the role of MBCs in pancreatic cancer.

## Conclusions

In conclusion, by exploring TME of pancreatic cancer using single-cell analysis, we found that MBCs were an important group of cells involved in cancer development of pancreatic cancer. The four-gene prognostic model based on markers of MBCs could predict overall survival and guide personalized therapy for pancreatic cancer patients. Importantly, we discovered two subgroups of MBCs (MBC_0 and MBC_1) with strong correlation with PAAD prognosis. Further studies are needed to explore the mechanism of MBC_0 and MBC_1 in PAAD progression.

## Data Availability Statement

The original contributions presented in the study are included in the article/**Supplementary Material**. Further inquiries can be directed to the corresponding author.

## Author Contributions

WH and XY conceptualized and designed the research; XY drafted the manuscript and got agreement to be accountable for all aspects of the work in ensuring that questions related to the accuracy or integrity of any part of the work are appropriately investigated and resolved; SZ and JL contributed to date acquisition; YL analyzed data; WH and XY interpreted data; XY revised the manuscript for important intellectual content. All authors contributed to the article and approved the submitted version.

## Conflict of Interest

The authors declare that the research was conducted in the absence of any commercial or financial relationships that could be construed as a potential conflict of interest.

## Publisher’s Note

All claims expressed in this article are solely those of the authors and do not necessarily represent those of their affiliated organizations, or those of the publisher, the editors and the reviewers. Any product that may be evaluated in this article, or claim that may be made by its manufacturer, is not guaranteed or endorsed by the publisher.

## Supplementary Material

Supplementary Figure 1.
**(A)** A work flow chart of this study. **(B, C)** Preprocessing of single-cell data. Quality control before **(B)** and after **(C)** preprocessing.

Supplementary Figure 2. Horizontal axis indicates samples. **(A)** Single cell counts of raw data and clean data. **(B, C)** Principle component analysis (PCA) of single-cell data. PCA plots before **(D)** and after **(E)** data preprocessing in GSE cohorts.

Supplementary Figure 3. The distribution of different clinical features and MBCs in IS1 and IS2.

Supplementary Figure 4. LASSO Cox regression analysis for optimizing the prognostic model. **(A)** The coefficients of each gene with the changing lambda value. Red dotted line indicates lambda = 0.0661. **(B)** 95% confidence interval of partial likelihood deviance of different lambda values. Red dot indicates lambda = 0.0661.

Supplementary Figure 5. Evaluation of the 4-gene prognostic model in GEO cohort. **(A)** The distribution of PAAD samples and expression of prognostic genes ranking by risk score. **(B)** ROC curve of the prognostic model in predicting 1-year, 3-year and 5-year overall survival. **(C)** Kaplan-Meier survival plot of high-risk and low-risk groups. Log-rank test was conducted.

Supplementary Figure 6. Evaluation of the 4-gene prognostic model in ICGC-AU cohort. **(A)** The distribution of PAAD samples and expression of prognostic genes ranking by risk score. **(B)** ROC curve of the prognostic model in predicting 1-year, 3-year and 5-year overall survival. **(C)** Kaplan-Meier survival plot of high-risk and low-risk groups. Log-rank test was conducted.

Supplementary Figure 7. Comparison of TME between high-risk and low-risk groups in GEO cohort. **(A)** A heatmap describing distribution of 22 immune cells in high-risk and low-risk groups. **(B)** Enrichment score of 10 oncogenic pathways in high-risk and low-risk groups. **(C)** Immune score of high-risk and low-risk groups. **(D–F)** Expression of immune checkpoints **(D)**, chemokines **(E)** and chemokine receptors **(F)** in two groups. Student t test was performed between two groups. ns, no significance. *P < 0.05, **P < 0.01, ***P < 0.001, ****P < 0.0001.

Supplementary Figure 8. **(A)** Association between IS1-2 and existing molecular subtypes and patients in high-risk and low-risk groups. **(B)** Interaction between four key genes. **(C)** Relationship between the expression of four key genes and memory B cells.

Supplementary Figure 9. **(A)** Markers of MBC_0, MBC_1 and MBC_2. **(B)** The distribution of three MBC subgroups in different pseudotime. **(C)** The distribution of seven markers in different pseudotime.

Supplementary Figure 10.
**(A)** Prognostic difference forest map of DDK1 in Pan cancer. **(B)** Prognostic difference forest map of ANLN in Pan cancer. **(C)** Prognostic difference forest map of SERPINB5 in Pan cancer. **(D)** Prognostic difference forest map of ARNTL2 in Pan cancer.
