# Supplementary figures and images for: Discovering a Four-Gene Prognostic Model Based on Single-Cell Data and Gene Expression Data of Pancreatic Adenocarcinoma
Source: Front Endocrinol (Lausanne). 2022 Jun 21;13:883548. doi: 10.3389/fendo.2022.883548 (PMC9253429; doi:10.3389/fendo.2022.883548)

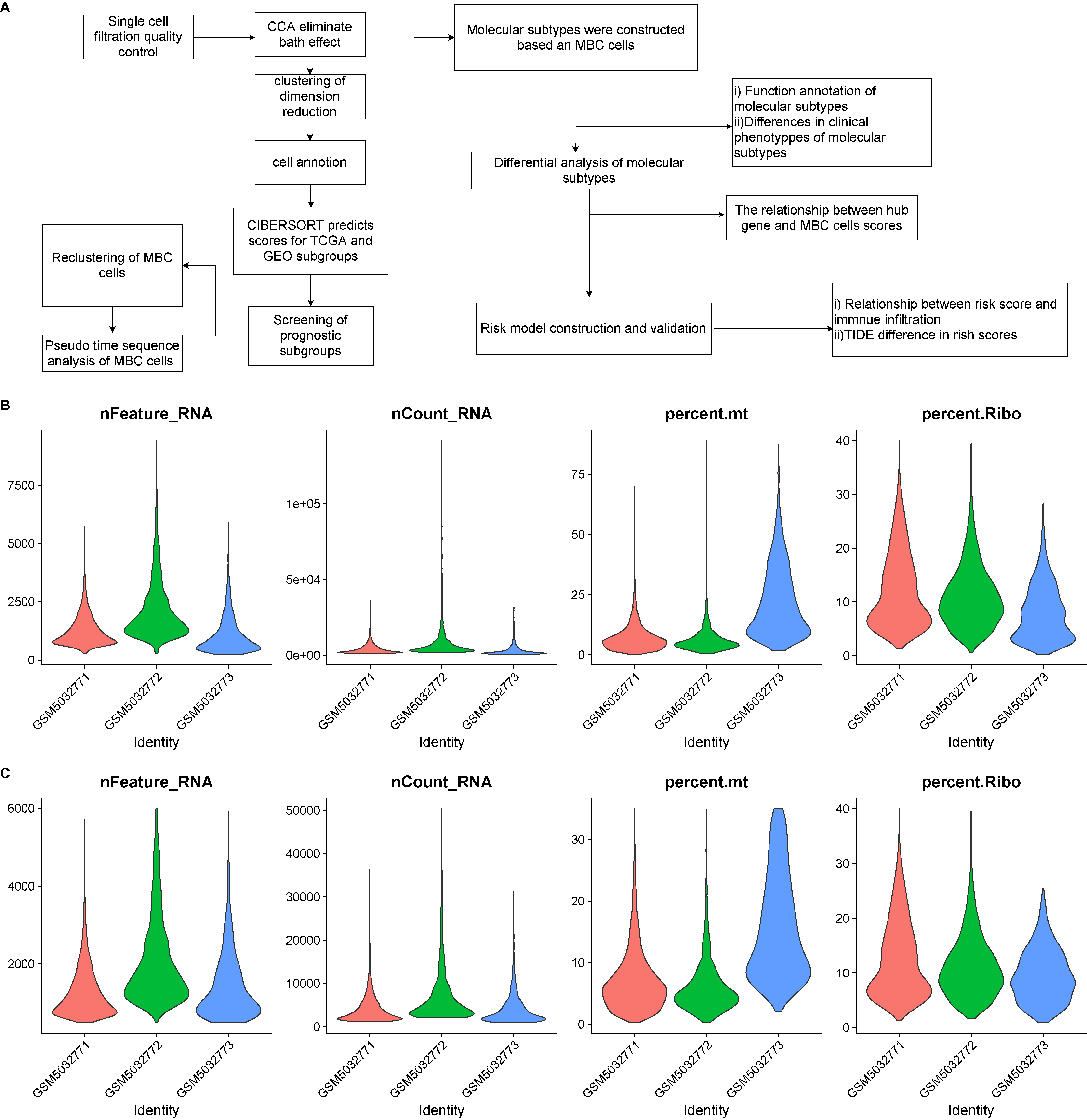

Supplement: Supplementary Figure 1. — (A) A work flow chart of this study. (B, C) Preprocessing of single-cell data. Quality control before (B) and after (C) preprocessing. [file Image_1.tif]

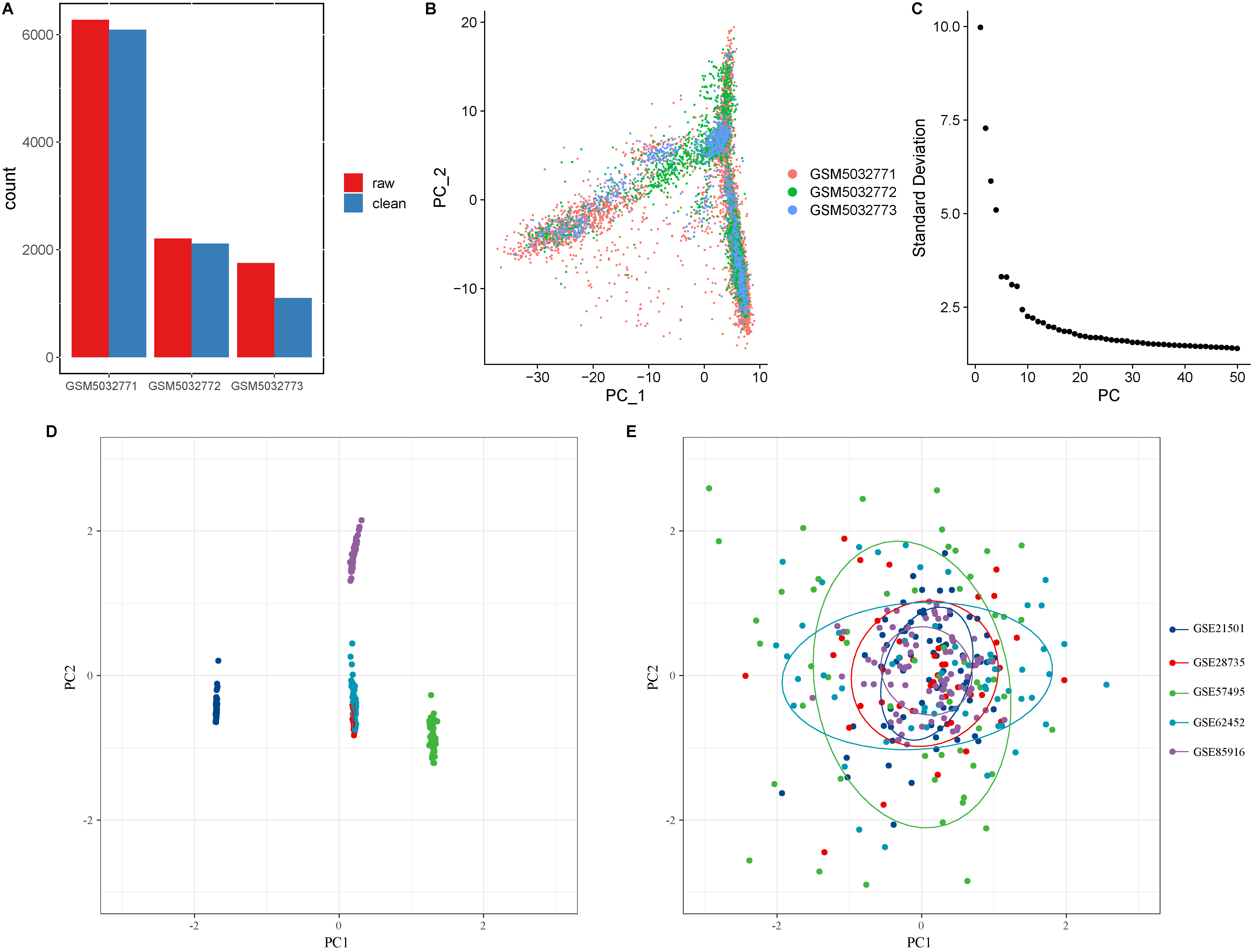

Supplement: Supplementary Figure 2. — Horizontal axis indicates samples. (A) Single cell counts of raw data and clean data. (B, C) Principle component analysis (PCA) of single-cell data. PCA plots before (D) and after (E) data preprocessing in GSE cohorts. [file Image_2.tif]

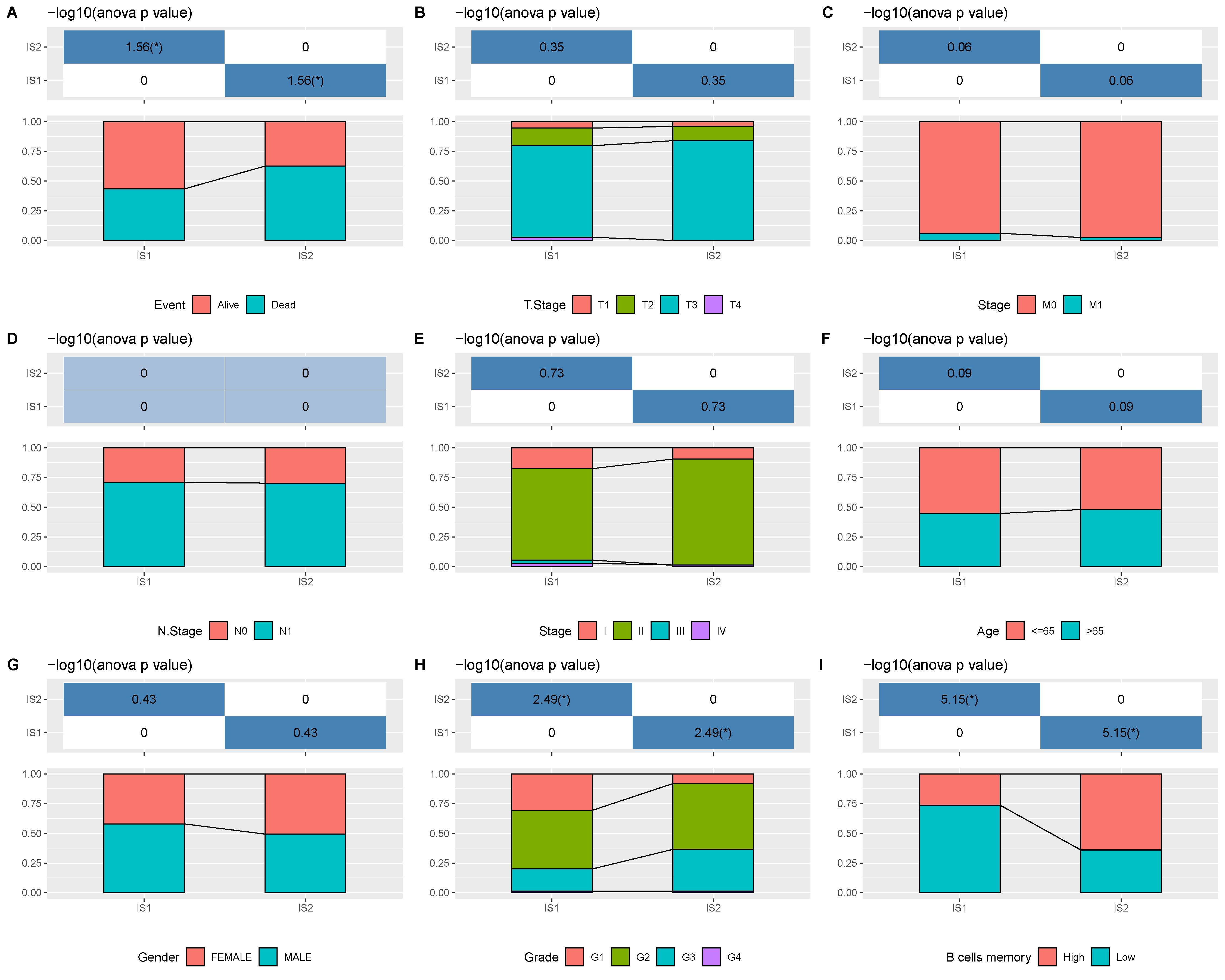

Supplement: Supplementary Figure 3. — The distribution of different clinical features and MBCs in IS1 and IS2. [file Image_3.jpeg]

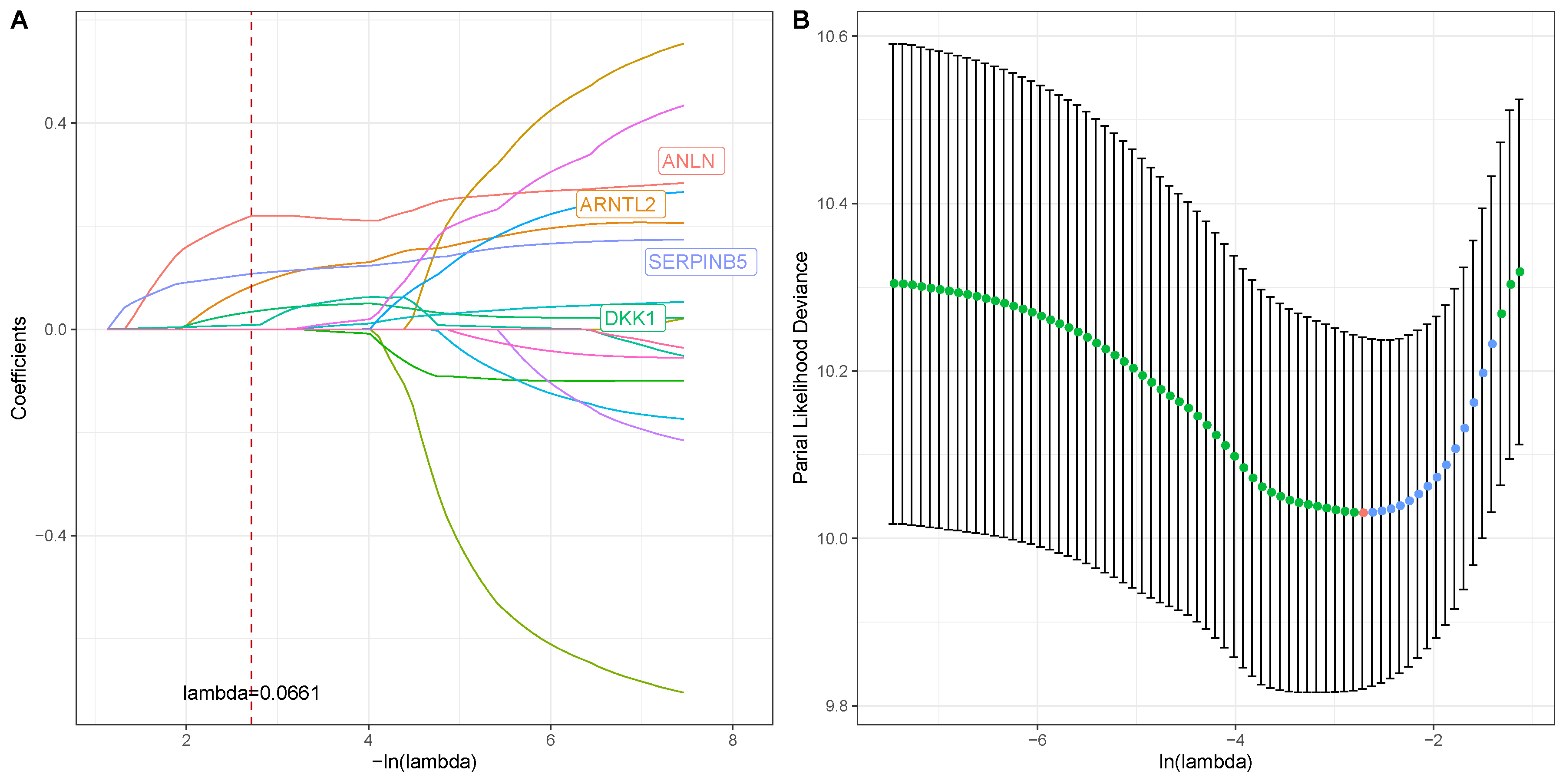

Supplement: Supplementary Figure 4. — LASSO Cox regression analysis for optimizing the prognostic model. (A) The coefficients of each gene with the changing lambda value. Red dotted line indicates lambda = 0.0661. (B) 95% confidence interval of partial likelihood deviance of different lambda values. Red dot indicates lambda = 0.0661. [file Image_4.jpeg]

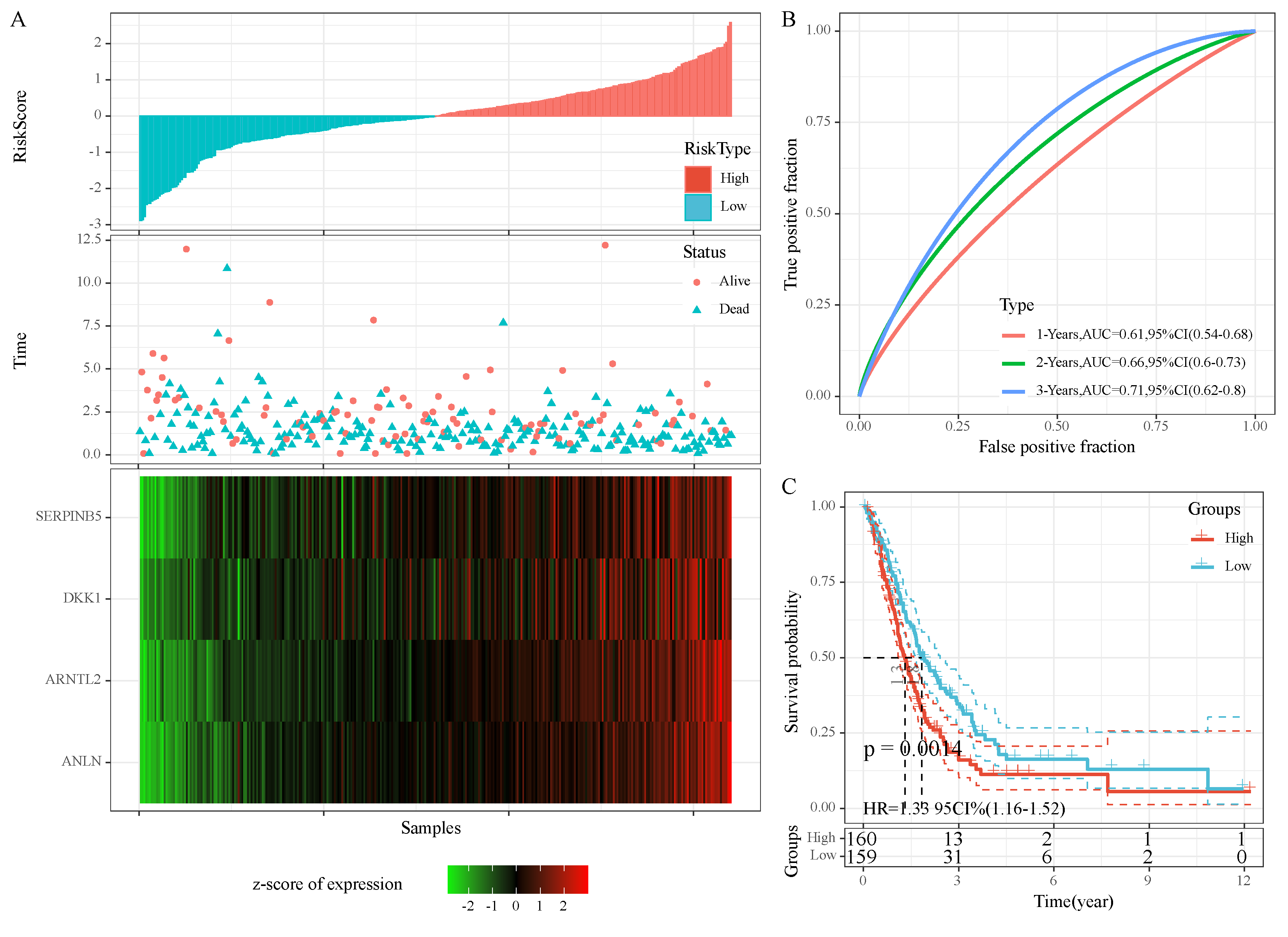

Supplement: Supplementary Figure 5. — Evaluation of the 4-gene prognostic model in GEO cohort. (A) The distribution of PAAD samples and expression of prognostic genes ranking by risk score. (B) ROC curve of the prognostic model in predicting 1-year, 3-year and 5-year overall survival. (C) Kaplan-Meier survival plot of high-risk and low-risk groups. Log-rank test was conducted. [file Image_5.tif]

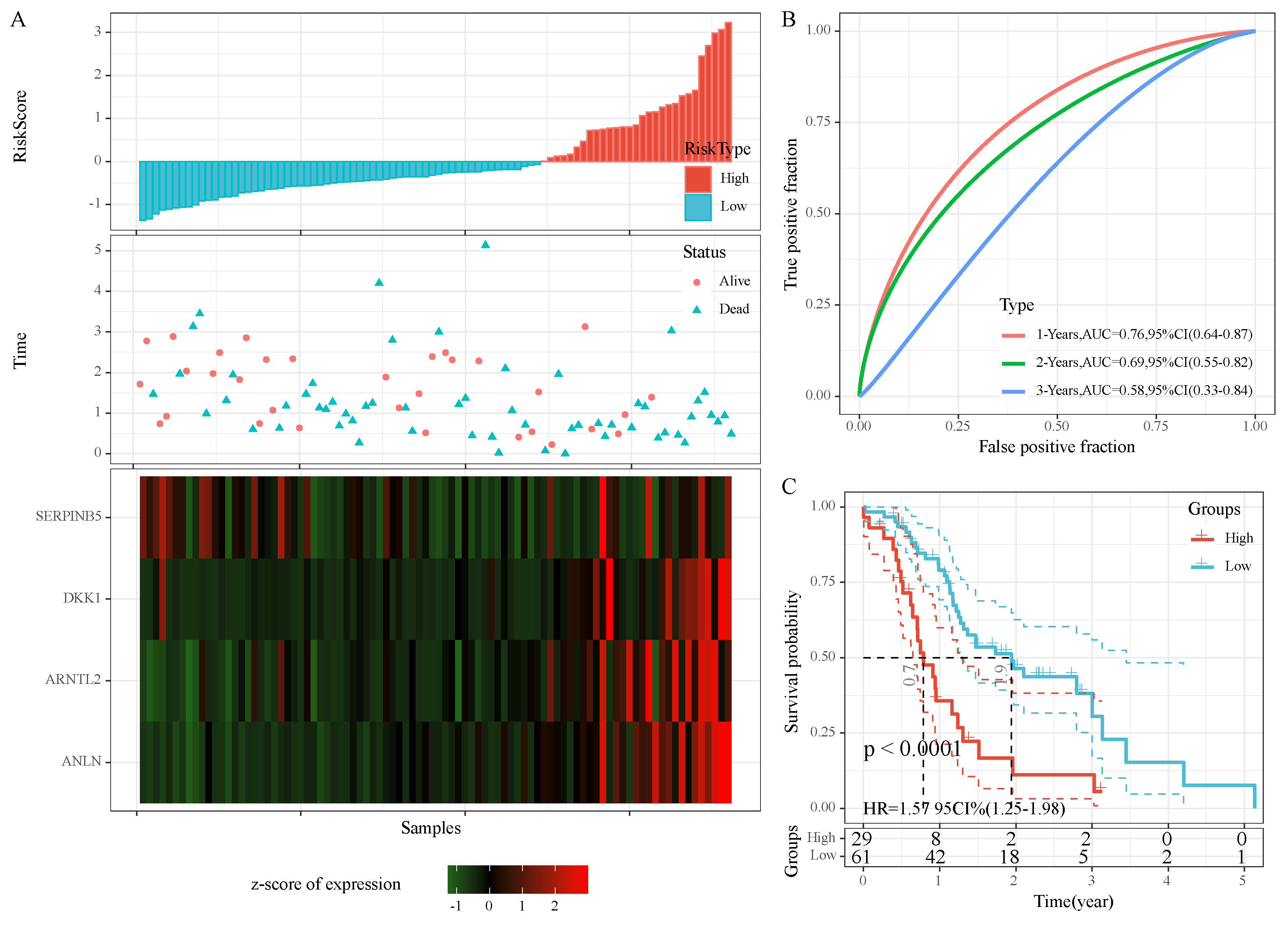

Supplement: Supplementary Figure 6. — Evaluation of the 4-gene prognostic model in ICGC-AU cohort. (A) The distribution of PAAD samples and expression of prognostic genes ranking by risk score. (B) ROC curve of the prognostic model in predicting 1-year, 3-year and 5-year overall survival. (C) Kaplan-Meier survival plot of high-risk and low-risk groups. Log-rank test was conducted. [file Image_6.tif]

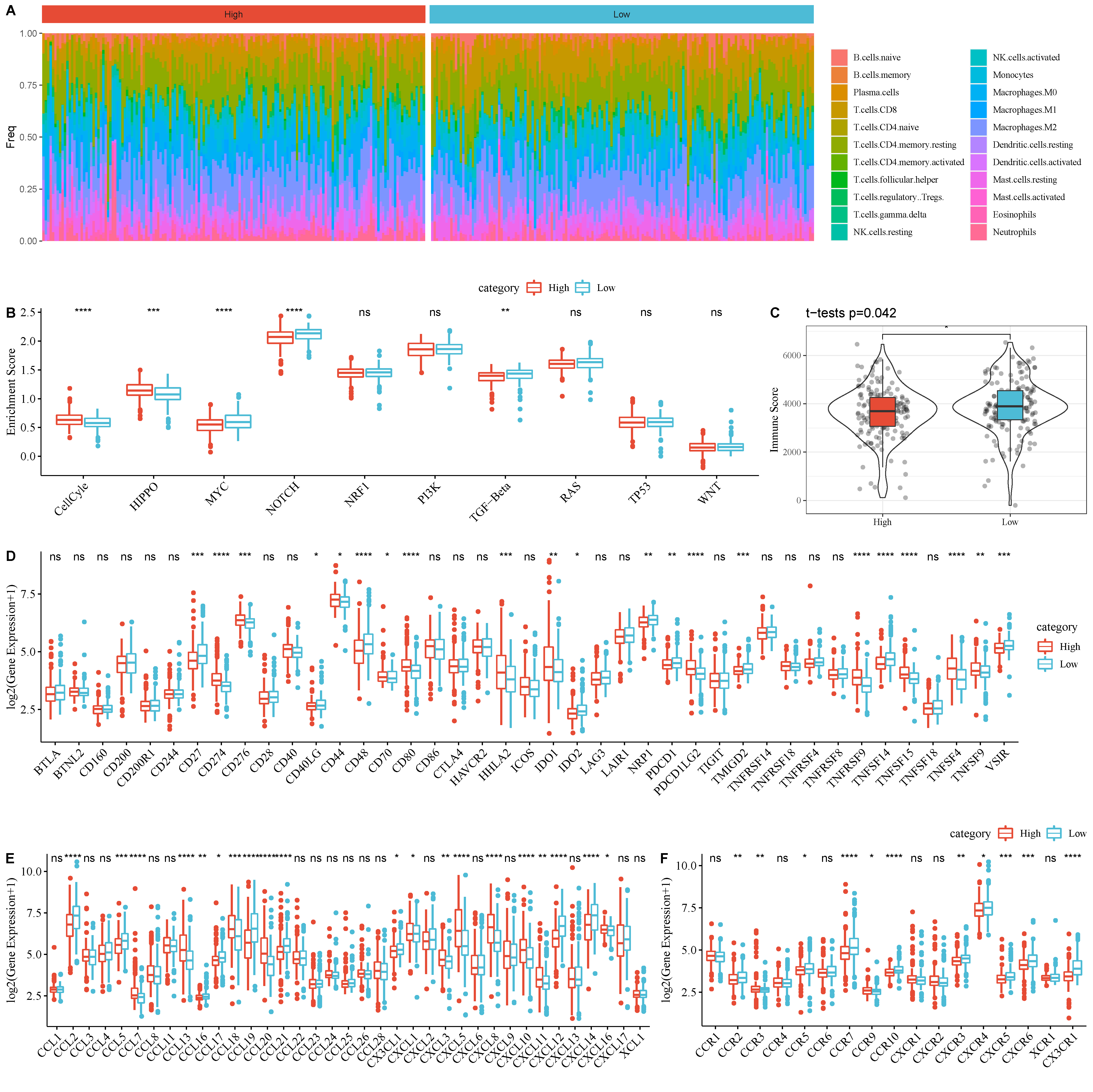

Supplement: Supplementary Figure 7. — Comparison of TME between high-risk and low-risk groups in GEO cohort. (A) A heatmap describing distribution of 22 immune cells in high-risk and low-risk groups. (B) Enrichment score of 10 oncogenic pathways in high-risk and low-risk groups. (C) Immune score of high-risk and low-risk groups. (D–F) Expression of immune checkpoints (D), chemokines (E) and chemokine receptors (F) in two groups. Student t test was performed between two groups. ns, no significance. *P < 0.05, **P < 0.01, ***P < 0.001, ****P < 0.0001. [file Image_7.jpeg]

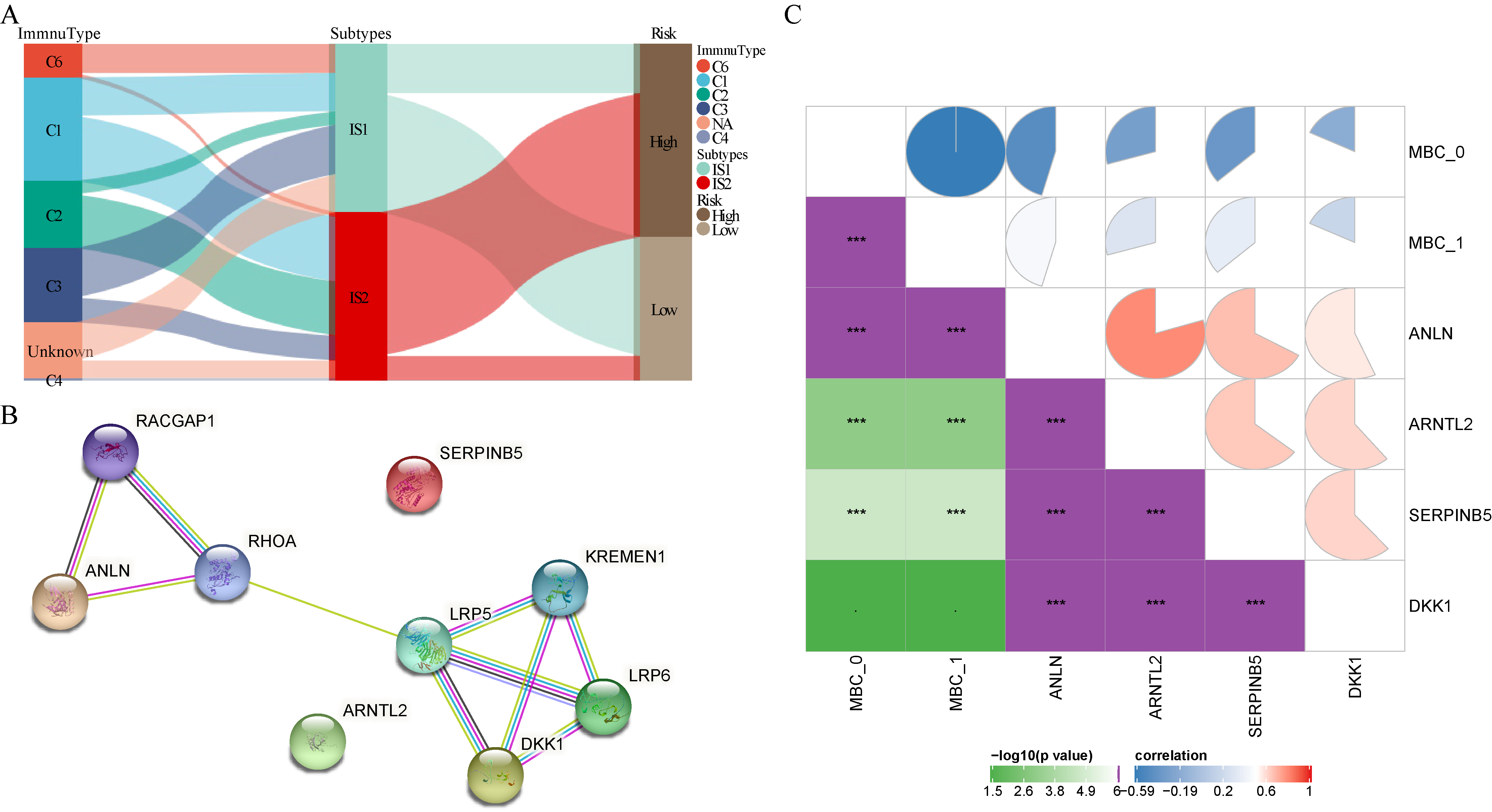

Supplement: Supplementary Figure 8. — (A) Association between IS1-2 and existing molecular subtypes and patients in high-risk and low-risk groups. (B) Interaction between four key genes. (C) Relationship between the expression of four key genes and memory B cells. [file Image_8.tif]

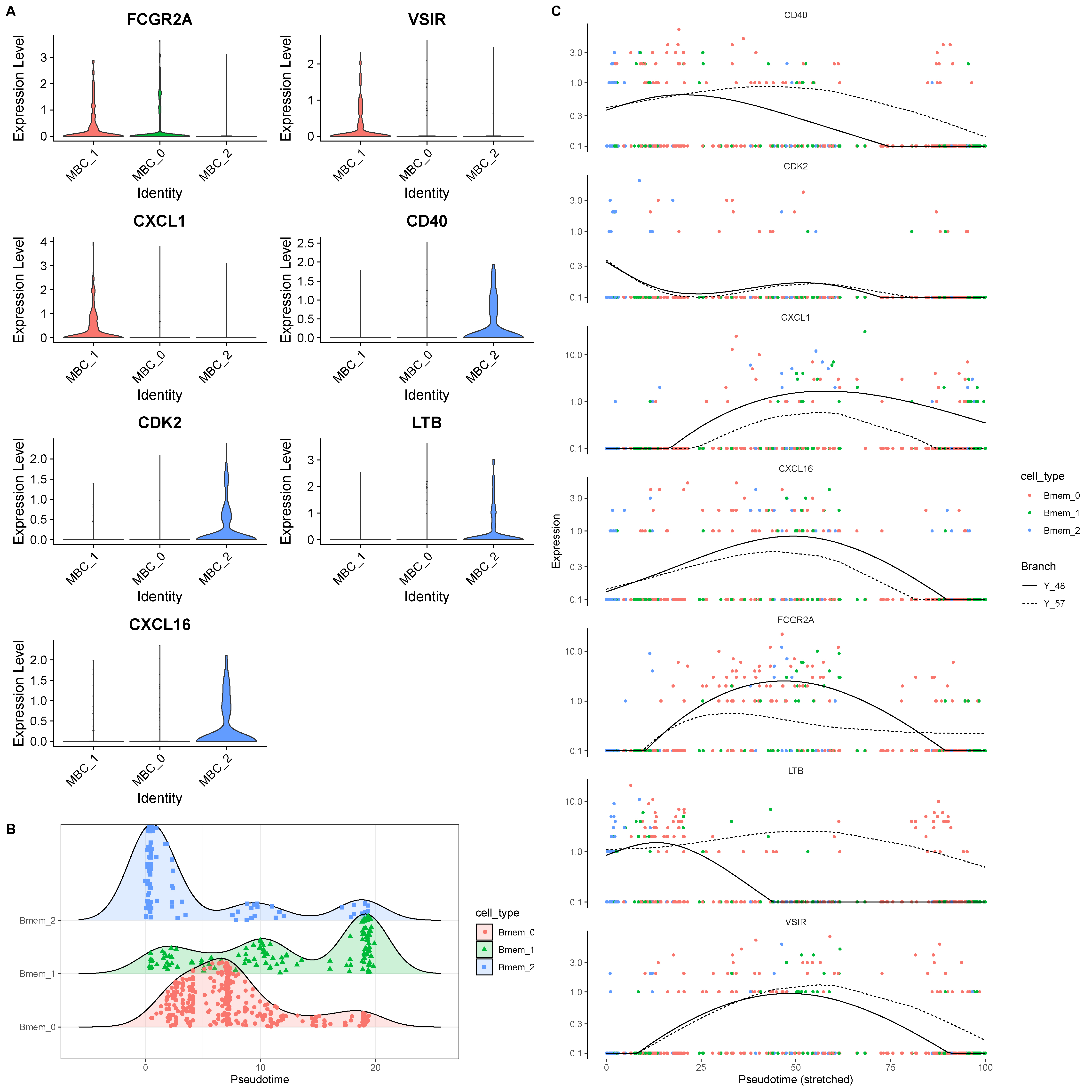

Supplement: Supplementary Figure 9. — (A) Markers of MBC_0, MBC_1 and MBC_2. (B) The distribution of three MBC subgroups in different pseudotime. (C) The distribution of seven markers in different pseudotime. [file Image_9.jpeg]

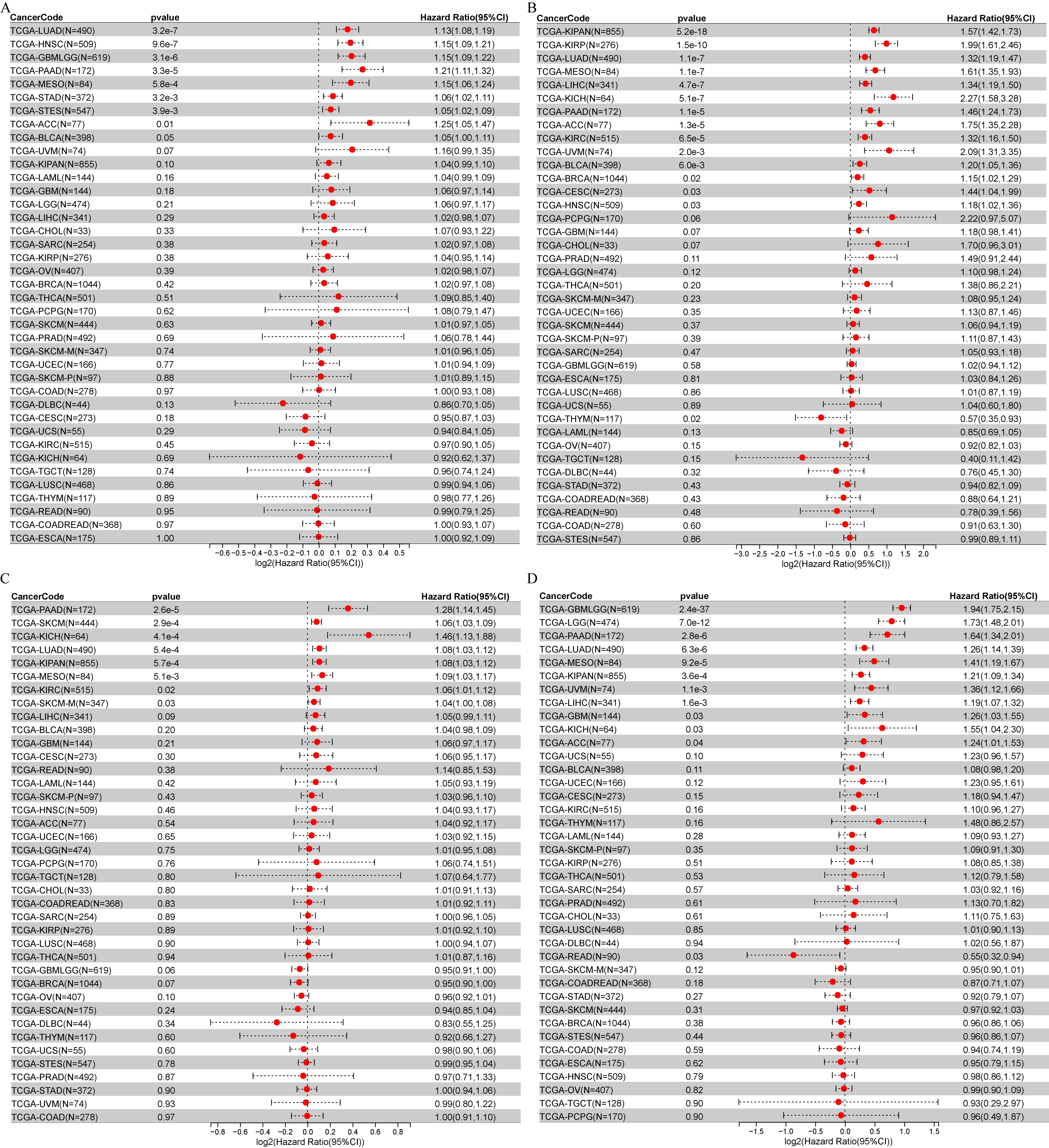

Supplement: Supplementary Figure 10. — (A) Prognostic difference forest map of DDK1 in Pan cancer. (B) Prognostic difference forest map of ANLN in Pan cancer. (C) Prognostic difference forest map of SERPINB5 in Pan cancer. (D) Prognostic difference forest map of ARNTL2 in Pan cancer. [file Image_10.tif]
