# Supplementary material for: Discovering a Four-Gene Prognostic Model Based on Single-Cell Data and Gene Expression Data of Pancreatic Adenocarcinoma
Source: Front Endocrinol (Lausanne). 2022 Jun 21;13:883548. doi: 10.3389/fendo.2022.883548 (PMC9253429; doi:10.3389/fendo.2022.883548)
Supplement: Supplementary file 11 [file Table_1.docx]

Supplementary TableS1: Sample clinical characteristics of each data set

|  | TCGA | ICGC | GEO |
| --- | --- | --- | --- |
| Gender |  |  |  |
| Female | 70 | 43 | NA |
| Male | 80 | 47 | NA |
| OS event |  |  |  |
| dead | 80 | 58 | 225 |
| alive | 70 | 32 | 94 |
| T |  |  |  |
| T1 | 7 | 1 | 1 |
| T2 | 20 | 8 | 9 |
| T3 | 119 | 73 | 60 |
| T4 | 2 | 1 | 1 |
| unknown | 2 | 7 | 248 |
| M |  |  |  |
| M0 | 70 | 3 | NA |
| M1 | 3 | 7 | NA |
| unknown | 77 | 80 | 319 |
| N |  |  |  |
| N0 | 43 | 26 | NA |
| N1 | 102 | 57 | NA |
| unknown | 5 | 7 | 319 |
| Stage |  |  |  |
| I | 20 | NA | 88 |
| II | 122 | NA | 94 |
| III | 2 | NA | 10 |
| IV | 3 | NA | 6 |
| unknown | 3 | 90 | 121 |
| Grade |  |  |  |
| G1 | 28 | 1 | 2 |
| G2 | 78 | 51 | 32 |
| G3 | 40 | 30 | 29 |
| G4 | 2 | 0 | 1 |
| unknown | 2 | 8 | 255 |
| Age |  |  |  |
| >65 | 69 | 50 | NA |
| <=65 | 81 | 39 | NA |
| unknown | 0 | 1 | 319 |
| Total | 150 | 90 | 319 |
